# Editorial: Eco-friendly fabrication of energy storage materials: from batteries to supercapacitors

**DOI:** 10.3389/fchem.2026.1894576

**Published:** 2026-06-10

**Authors:** Min Hong, Chunrong Ma, Gang Chen

**Affiliations:** 1 Walker Department of Mechanical Engineering, Materials Science and Engineering Program & Texas Materials Institute (TMI), The University of Texas at Austin, Austin, TX, United States; 2 College of Physics, Qingdao University, Qingdao, Shandong, China; 3 School of Information and Control Engineering, China University of Mining and Technology, Xuzhou, Jiangsu, China

**Keywords:** artificial intelligence, battery recycling, green fabrication, interfacial engineering, sustainable energy storage

The growing demand for sustainable electrochemical energy storage has accelerated research into environmentally friendly fabrication strategies for batteries and supercapacitors. This Research Topic, Eco-Friendly Fabrication of Energy Storage Materials: From Batteries to Supercapacitors, highlights emerging approaches integrating green synthesis, advanced manufacturing, artificial intelligence (AI), and sustainable recycling technologies to enable next-generation energy systems. The contributions not only showcase recent breakthroughs but also outline a roadmap for addressing the environmental footprint of energy storage technologies—a critical prerequisite for achieving global carbon neutrality and circular economy goals.

A prominent theme among the collected articles is the integration of AI and machine learning (ML) into sustainable material design and energy system optimization. Abbaszadeh et al. presented an AI-driven microalgae-based bioenergy and green fabrication framework, demonstrating how artificial neural networks (ANNs) and support vector machines (SVMs) can optimize biomass conversion, pollutant removal, and renewable energy material production. Importantly, the work highlighted microalgal biomass as a renewable precursor for carbon-based electrode materials applicable in batteries and supercapacitors, while simultaneously supporting circular economy concepts through biowaste valorization. Looking forward, the integration of AI with biorefinery workflows could further reduce trial-and-error experimentation, although challenges remain in model generalizability across different algal strains and cultivation scales.

Similarly, Punna et al. explored AI-integrated manufacturing for advanced energy materials and catalytic systems. Their study demonstrated that ML-assisted frameworks can significantly accelerate catalyst discovery, reduce reliance on computationally expensive density functional theory (DFT) simulations, and optimize catalytic materials for hydrogen evolution, CO_2_ reduction, and ammonia synthesis. Furthermore, the integration of surrogate models, automated experimentation, and transfer learning established a closed-loop computational framework for sustainable material development. A key future direction will be the development of interpretable AI models that not only predict performance but also reveal underlying physicochemical mechanisms—thereby bridging the gap between data-driven screening and rational material design. Together, these two contributions emphasize the rapidly expanding role of AI in enabling low-carbon, resource-efficient material fabrication and energy system optimization.

Beyond computational approaches, advanced fabrication technologies for miniaturized energy storage devices also emerged as a key direction. Li et al. summarized recent progress in laser fabrication of MXene-based planar micro-supercapacitors. The review demonstrated how laser ablation, laser cutting, and laser modification technologies enable ultrafast maskless patterning, flexible device assembly, and microstructure engineering for MXene electrodes. Representative studies revealed strong correlations between laser processing strategies, device architectures, and electrochemical performance, highlighting scalable manufacturing opportunities for flexible and wearable electronics. The work illustrates how precise, solvent-minimized laser processing can support environmentally benign fabrication of next-generation micro-energy storage systems. Future efforts should focus on upscaling these laboratory-scale laser processes to roll-to-roll manufacturing, as well as expanding the material library beyond MXenes to include other two-dimensional materials and composites, while maintaining low energy consumption and chemical waste reduction.

Interfacial engineering for safer high-energy batteries was another important focus of this Topic. Xu et al. reported a conformal LATP coating strategy for Ni-rich NCM811 cathodes using a facile wet-chemical method. The amorphous LATP interfacial layer effectively suppressed side reactions, reduced transition-metal dissolution, and improved cycling stability. More importantly, the coating significantly enhanced thermal safety by increasing thermal runaway trigger temperatures and reducing maximum runaway temperatures under abuse conditions. This work demonstrates that eco-friendly interfacial engineering can simultaneously improve electrochemical performance and intrinsic battery safety, addressing a critical challenge for high-energy lithium-ion batteries. A promising avenue for future research is the development of adaptive or self-healing interfacial layers that can dynamically respond to degradation processes, thereby extending battery lifetime under real-world operating conditions. Moreover, combining such interfacial designs with AI-guided electrolyte formulation could unlock synergistic safety enhancements.

Sustainable battery recycling and regeneration technologies also constituted a major research direction. Wu et al. systematically reviewed microwave-assisted technologies for recycling spent lithium-ion batteries. Unlike conventional heating methods, microwave processing enables rapid volumetric heating with high energy efficiency and accelerated reaction kinetics. The review highlighted microwave-assisted pretreatment, carbothermal reduction, hydrometallurgical leaching, and direct regeneration of degraded electrode materials, including graphite and cathodes. Importantly, microwave technologies offer a promising low-carbon pathway toward closed-loop battery recycling and sustainable resource recovery. Looking ahead, the integration of microwave recycling with real-time monitoring and AI-based process control could further improve material recovery rates and reduce energy variability. An equally critical challenge is the design of batteries with recycling in mind from the outset—a concept often termed “design for recycling”—which would synergize with the microwave-assisted approaches reviewed here.

Collectively, the articles in this Research Topic reveal several emerging trends in sustainable energy storage materials. First, AI and ML are becoming increasingly important for accelerating material discovery, process optimization, and lifecycle management. Second, scalable green fabrication technologies such as laser processing and microwave heating are enabling low-energy and environmentally friendly manufacturing routes. Third, interfacial engineering and recycling strategies are increasingly integrated into the broader sustainability framework of electrochemical energy systems.

Overall, this Topic highlights the growing convergence of green chemistry, intelligent manufacturing, advanced interface science, and circular economy principles in modern energy storage research. We hope these contributions will inspire further interdisciplinary efforts toward environmentally responsible, scalable, and high-performance energy storage technologies.

Finally, we sincerely thank all authors, reviewers, and editors for their valuable contributions and support in making this Research Topic successful.

